# Redefining the limits of high-energy lithium batteries

**DOI:** 10.1093/nsr/nwaf513

**Published:** 2025-11-18

**Authors:** Jing Li, Hongtao Qu, Bao-Lian Su

**Affiliations:** Department of Chemistry, Laboratory of Inorganic Materials Chemistry (CMI), University of Namur, Belgium; Department of Chemistry, Laboratory of Inorganic Materials Chemistry (CMI), University of Namur, Belgium; Department of Chemistry, Laboratory of Inorganic Materials Chemistry (CMI), University of Namur, Belgium; State Key Laboratory of Advanced technology for Materials Synthesis and Processing, Wuhan University of Technology, China

Polymer electrolytes hold significant promise for high-energy lithium batteries due to their combination of safety and design flexibility. Particularly when paired with lithium-rich manganese-based layer oxide (LRMO) cathodes and anode-free systems, they are expected to achieve energy densities exceeding 600 Wh kg^−1^ [1]. However, the irreversible anionic reactions at the interface between LRMO and polymer electrolytes can trigger oxygen release and catalytic electrolyte decomposition, leading to severe interfacial degradation and poor cycling stability [2]. Therefore, mitigating the interfacial instability is a critical challenge for achieving high-energy lithium batteries. Current research efforts primarily focus on enhancing interfacial stability through strategies such as salt additives, ionic liquids and surface coating [3,4]. However, these approaches often tackle the symptoms rather than the root causes, making it challenging to simultaneously optimize energy density, interfacial chemistry and overall safety.

**Figure 1.**
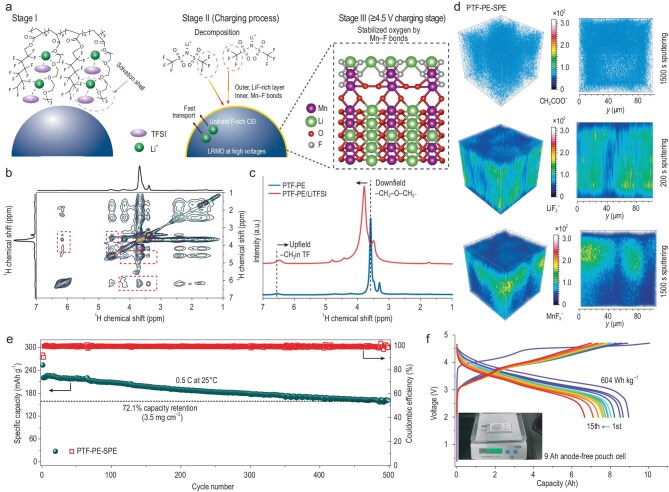
(a) Schematic diagram of FPE–SPE improving the interfacial stability of LRMO cathodes. Stage I is the *in situ* FPE-SPE with anion-rich solvation; stage II is anion decomposition at the charging process; and stage III is Mn–F bonds suppressing O_2_ escape. (b) 2D ^1^H–^1^HNOESY NMR spectrum of PTF–PE/LiTFSI. (c) ^1^H solid-state NMR spectrum of PTF–PE and PTF–PE/LiTFSI. (d) TOF-SIMS 3D and 2D tomography of the LRMO cathodes after 100 cycles using PTF–PE–SPE. (e) Long-term cycling of LRMO-based lithium metal batteries using PTF–PE–SPE at 0.5 C. (f) The voltage profiles of a 9 Ah anode-free pouch cell from the 1st to the 15th cycles at 0.05 C. Adapted with permission from Huang *et al*. [5].

Writing in *Nature*, Huang and colleagues proposed a solvation-molecular-level breakthrough to intrinsically regulate the interface of high-energy lithium batteries [5]. By engineering an innovative anion solvation structure construction strategy, they developed an in-built fluoropolyether-based quasi-solid-state polymer electrolyte (FPE–SPE). The electrolyte is copolymerized from weakly solvating fluorocarbon side chains, poly-(2,3,3,4,4,5,5,6,6,7,7-dodecafluoroheptyl acrylate) (PTF) and strongly solvating polyether backbones of polyethylene glycol methyl ether acrylate (PE). This molecular configuration induces the formation of an anion-rich solvation shell, in which the TFSI^−^ anions preferentially form contact ion pairs and aggregates at the LRMO interface, ultimately leading to the formation of a stable inorganic fluorine-rich cathode–electrolyte interphase (CEI). This CEI configuration generates an LiF outer layer that facilitates Li^+^ ion migration and interfacial kinetics, and an inner layer with Mn–F bonds that suppresses oxygen overoxidation and structure phase transitions, thereby supporting the realization of unprecedented high-energy lithium batteries (Fig. 1a).

The 2D ^1^H–^1^H nuclear Overhauser enhancement spectroscopy (NOESY) NMR spectrum reveals that the addition of LiTFSI into PTF–PE induces distinct cross-peaks between fluorohydrocarbon protons (–CH_2__–_CF_2_–/CHF_2_) and ether protons (–O–CH_2_) in the polyether chains (Fig. 1b), indicative of interchain –F···Li^+^···O–CH_2_^−^ interaction [6]. Correspondingly, the ^1^H solid-state NMR spectrum exhibits downfield shifts for ether protons and upfield shifts for fluorohydrocarbon protons (Fig. 1c), confirming the interaction between Li^+^ ions and –CF_2_ fluorine atoms. This –F···Li^+^···O–CH_2_^−^ coordination provides a stable Li⁺ conduction pathway and drives the formation of a TFSI^−^ anion-rich solvation shell, optimizing ionic conductivity of polymer electrolyte and interfacial dynamics. Time-of-flight secondary ion mass spectrometry (TOF-SIMS) further demonstrates that LiF_2_^−^ species tend to accumulate in the surface layer of the LRMO after charging, while MnF_3_^−^ species are observed on the subsurface. This observation suggests that the O atoms in the lattice of the LRMO surface are partially substituted by F atoms to build the robust Mn–F bonds, which tune oxygen redox, trap the oxidized lattice oxygen O_2_^n−^ species, and suppress the O_2_ release, ultimately enhancing both specific capacity and cycling stability.

Based on these findings, the PTF–PE–SPE exhibits remarkable interfacial stability, supporting highly reversible oxygen redox in LRMO cathodes. The Li|PTF–PE–SPE|LRMO cell maintains a capacity retention of 72.1% with an average Coulombic efficiency of 99.5% after 500 cycles at 0.5 C (Fig. 1e). The robust interfacial compatibility of PTF–PE–SPE also makes it suitable for commercial LiNi_0.8_Co_0.1_Mn_0.1_O_2_ cathodes, achieving 90% capacity retention after 200 cycles. Encouragingly, the Cu|PTF–PE–SPE|LRMO pouch cell achieves a discharge capacity of 8.96 Ah and a high initial specific energy of 604 Wh kg^−1^/1027 Wh L^−1^ at 0.05 C based on the total weight and volume of the pouch cell, and maintains a discharge capacity of 6.66 Ah after 15 cycles at 25°C (Fig. 1f). The measured value of specific energy achieved in this work surpasses those of state-of-the-art lithium metal pouch cells employing polymer or inorganic electrolytes [7,8]. Furthermore, the PTF–PE–SPE demonstrates high-safety performance for the high-energy lithium batteries. The combination of the flame-retardant nature of fluorinated polyether and a stable interfacial layer significantly raises the thermal runaway onset temperature compared with conventional lithium batteries, providing critical safety assurance for large-scale applications of solid-state batteries. Although PTF–PE–SPE exhibits excellent interfacial stability and high energy density in the laboratory, key challenges remain for practical application, including scalable and cost-effective synthesis, mechanical durability during cycling, compatibility with high-loading electrodes, and long-term stability under realistic operating conditions.

This work achieves a molecular-level design of polymer electrolytes by constructing an anion-rich solvation structure that spontaneously forms a fluorine-rich interfacial layer. This strategy resolves the long-standing trade-off between ionic conductivity and interfacial stability, enabling the practical application of high-voltage lithium-rich cathodes and anode-free batteries. Beyond its record electrochemical performance, this work represents a paradigm shift in electrolyte design, moving from conventional external modification strategies like additives, coating and fillers, to intrinsic interface regulation governed by solvation chemistry. Moreover, the anion-rich solvation concept holds promise for broader applicability, including stabilizing high-voltage cathodes like high-nickel LiNi_0.8_Co_0.1_Mn_0.1_O_2_ and other lithium-rich materials, as well as diverse anode systems such as silicon-based anodes, by promoting robust solid–electrolyte interphase (SEI)/CEI formation and mitigating interfacial degradation. When we discuss range anxiety, this molecular-level innovation is redefining the limits of high-energy lithium batteries.
